# Editorial: AI for health behavior change

**DOI:** 10.3389/fdgth.2025.1735279

**Published:** 2025-12-11

**Authors:** Nataliya Mogles, Sian Joel-Edgar, Chao Zhang, Michel Klein

**Affiliations:** 1 Northeastern University London, London, United Kingdom; 2 Technische Universiteit Eindhoven, Eindhoven, Netherlands; 3 Department of Computer Science, Vrije Universiteit Amsterdam, Amsterdam, Netherlands

**Keywords:** AI, digital health, behavior change, responsible AI, digital coaching, hybrid AI applications

## Introduction

In spite of advances in medicine and pharmacology, a healthy lifestyle as a means of chronic disease prevention is gradually acquiring more focus and attention, as prevention takes substantially less resources than treatment. It often requires enormous behavioural efforts from individuals when they have to break their routine and well-established patterns of behaviour in the context of exercising and a healthy diet, for example. Formation of new habits in these aspects needs constant coaching, monitoring and support. With the advances in technology and evolution of various relatively low cost monitoring devices for health-related variables such as movement, heart rate, and blood pressure, along with advances in Artificial Intelligence (AI), it has become common to deploy these technologies to solve the problem of introducing healthy lifestyles and to offer 24-hour support for behaviour change.

The first digital health applications started to develop two decades ago under the terms mHealth or eHealth (1). Fogg was a pioneer of the first behavior change digital applications for healthy lifestyle and coined the “persuasive technology” term in 2002 (2). Now with the recent advances in AI exemplified by deep learning and large language models, there is again a growing interest in applying the newer generation of tools to digital health (3) and specifically to health behavior change (4, 5). However, the application of digital technologies and AI to health behaviour change remains a challenging and complex multi-faceted task with many disciplines and stakeholders involved.

This special issue reflects challenges and perspectives on the current research and design of AI-based digital systems for health-related behavior change. We deliberately did not specify where the boundaries of AI or not AI-based digital interventions lie since the concept of AI is not well-defined and depends on new technological advancements and fashion that emerge in a society during a specific time frame. The collection comprises contributions from different institutions across six countries: Switzerland, Sweden, Ireland, Netherlands, United States and Finland.

Hao et al., in their perspective article on research challenges for hybrid human AI in lifestyle and behavior change support, describe challenges of development and deployment of human-centred health support digital systems. Among others, the authors point out ethical considerations that arise from the design of health support applications. Nowadays, digital health systems still deploy exclusive rather than inclusive design since the majority of these applications are being tested and used by highly educated and higher income individuals. The authors argue that in order to address diversity, equity and inclusion, health information systems development should be a truly interdisciplinary process with detailed case studies and pilot studies, along with collaborations between various stakeholders and practitioners from different fields. The authors state that there is still a long time needed to bring research on such support systems to their deployment in people's everyday lives.

Loughnane et al. present the findings of a systematic review that explores different types of coaching in digital health interventions and their lifestyle outcomes and engagement: human, AI-based and hybrid health coaching. The authors identified and synthesised thirty-five relevant studies with a focus on healthy lifestyle coaching. The authors point out that most of these studies were exploratory and not consistent in terms of outcome measures; they had diverse intervention designs and coaching characteristics, which pose challenges in comparative analysis. The authors of the review conclude that AI coaching could be very effective for more specific, narrow and structured tasks like goal-setting, but when it comes to building relationships and working connections with a coach, it lacks the depth which is crucial for long-term behavioral change. For example, AI coaching demonstrated more positive outcomes in exercising and physical activity behaviors, while human-facilitated coaching exhibited better results for psychological wellbeing and stress management support.

Švihrová et al. present a framework for the design of digital health interventions based on causal inference and Bayesian multi-armed bandits(MAB) approach from reinforcement learning. The authors discuss implications for the design of adaptive behavior change interventions that analyse contextual data in real time. This method can be used to infer the user's behavior for a tailored dynamic feedback. The authors argue that adaptive mHealth interventions with MAB algorithms represent a promising approach for tailored behavior change. It is pointed out, however, that the success of this approach depends on careful evaluation, psychological insight, truly interdisciplinary collaboration and strict adherence to regulatory and ethical standards.

Lindgren et al. present their original research where they apply a responsible AI participatory design process during the development of an AI system for health behavior change as part of the STAR-C digital coach project. The authors adopt a responsible AI framework and emphasise the importance of human agency and inclusion of human values in the design of AI systems for behavior change. In most commercial digital health applications, users and other stakeholders do not participate in the design of this technology. Participatory design is crucial in this context of complex socio-technical systems when AI applications exhibit emergent behavior which cannot easily be explained and adjusted. The authors' findings indicate that users would expect the system and a digital coach for health behavior change to present hard facts and be more proactive.

The current special issue demonstrates that there is a very promising potential in the development and applications of AI-based digital behaviour change systems for healthy lifestyle, though this potential is not fully realised yet, and a lot of research is still needed both on the design of such systems and on their complex interaction with users. The main challenge now lies in the plane of making such systems more humane and more ethical. True success in this area might evolve over decades, also with the development of more intelligent, more humane, less invasive and even cheaper technologies affordable for and adapted to all users across the globe.
